# Endoscopic CO(2) Laser Horizontal Partial Laryngectomy in Larynx Carcinosarcoma

**DOI:** 10.1155/2014/278640

**Published:** 2014-07-14

**Authors:** Andrea Ciorba, Chiara Bianchini, Valeria Iannini, Antonio Faita, Enzo Bianchini, Francesco Stomeo, Stefano Pelucchi, Antonio Pastore

**Affiliations:** ^1^ENT and Audiology Department, University Hospital of Ferrara, Via Aldo Moro, Cona, 8 44124 Ferrara, Italy; ^2^Pathology Section, ENT and Audiology Department, University Hospital of Ferrara, Italy

## Abstract

*Background.* Carcinosarcoma is an extremely rare malignant neoplasm, with both a malignant epithelial and mesenchymal component, that rarely affects the larynx. *Aim.* Aim of this paper is to describe the case of a patient affected by a larynx carcinosarcoma treated by endoscopic horizontal partial laryngectomy with CO(2) laser and particularly discuss the histogenetic hypothesis as well as the possible treatment modalities of this rare lesion. *Methods.* Case report and literature review. *Discussion and Conclusion.* Still little is known about the biology of carcinosarcoma and there is still no consensus in the literature on the treatment of these tumors. Endoscopic horizontal partial laryngectomy could represent another treatment option in selected cases.

## 1. Introduction

Carcinosarcoma is a malignant tumor with both a malignant epithelial and mesenchymal component. Most reported cases occurred in the major salivary glands; other reported sites are larynx and pharynx and, less frequently, oral and nasal cavities and esophagus [1–5].

Carcinosarcoma of larynx and hypopharynx represents less than 1% of all malignant tumours [6]. Reported risk factors are those of squamous cell carcinoma (i.e., smoking and alcoholism), as well as exposure to radiation. The classic clinical presentation features of the larynx carcinosarcoma are dysphonia and eventually dyspnea due to laryngeal obstruction [7].

The histogenesis of carcinosarcoma is controversial: its origin has been ascribed to the differentiation of primitive blastic mesenchymal cells. At diagnosis, a true carcinosarcoma exhibits a malignant mesenchymal component (sarcoma), as well as an epithelial component (carcinoma or adenocarcinoma). The histologic exam often reveals the presence of pleomorphic cells and atypical mitosis.

Most of the cases of laryngeal carcinosarcoma have been treated with laryngectomy. Only in few cases organ preservation has been achieved by partial laryngectomy with external cervical approach [8, 9].

In this paper we describe a case of larynx carcinosarcoma treated by endoscopic laser CO(2) horizontal partial laryngectomy.

## 2. Case Report

A 61-year-old woman complaining from dysphagia for 6 months as well as dysphonia and dyspnoea for 2 months was referred to the ENT Department at the University Hospital of Ferrara in April 2012. The patient also reported weight loss of about 5-6 pounds in the last months before. She had a history of smoking, hypertension, diabetes, hypercholesterolemia, vascular encephalopathy, and multiple vascular diseases. In particular, she underwent bilateral carotid arteries thromboendarterectomy. She also had vascular surgery for aortic dissection in the previous years; the last CT angiographies also showed a dissection of the left common carotid artery (with severe lumen reduction) and several calcified atheromatic lesions of thoracic and abdominal aorta.

The ENT evaluation disclosed the presence of an epiglottic laryngeal face neoformation. No lymph nodes were detectable clinically at the neck inspection. The remaining head and neck exam was unremarkable.

The patient underwent head, neck, and chest contrast CT scans that disclosed the presence of a lesion, with inhomogeneous contrast enhancement of a transverse diameter of 25 × 17 mm and a craniocaudal extension of 22 mm, in correspondence to the laryngeal side of the epiglottis. The scan also showed a left submandibular lymph node of 10 × 14 mm, as well as some bilateral jugular and subcentrimetic digastric lymph nodes with reactive features. Nonetheless, the fine needle aspiration cytology (ultrasound-guided) of the lymph nodes revealed lymphocytes at different stages of maturation and no neoplastic cells.

A biopsy under local anaesthesia was then performed. Since the histological examination disclosed the malignant nature of the lesion, and considering the important comorbidities of the patient, she was evaluated by a multidisciplinary team (composed of the ENT surgeon, the vascular surgeon, the anaesthesiologist, the oncologist, and the radiation therapy oncologist), in order to establish the best possible treatment. It was therefore decided to perform a microlaryngoscopy in order to evaluate the possibility of an endoscopic surgical excision of the laryngeal tumour, while at the same time it was established not to proceed with the lymph node prophylactic dissection in the same surgical session due to (i) the complex and severely compromised situation of the neck vasculature; (ii) the absence of clinical, radiological, and cytological evidence of lymph node involvement.

Under general anaesthesia and after tracheotomy, a microlaryngoscopy was then performed; since it confirmed the possibility of an endoscopic excision, an endoscopic CO(2) laser horizontal partial laryngectomy was performed.

During anaesthesia the patient also underwent an esophageal-gastro-duodenoscopy without evidence of lesions in all the examined districts.

**Figure 1:**
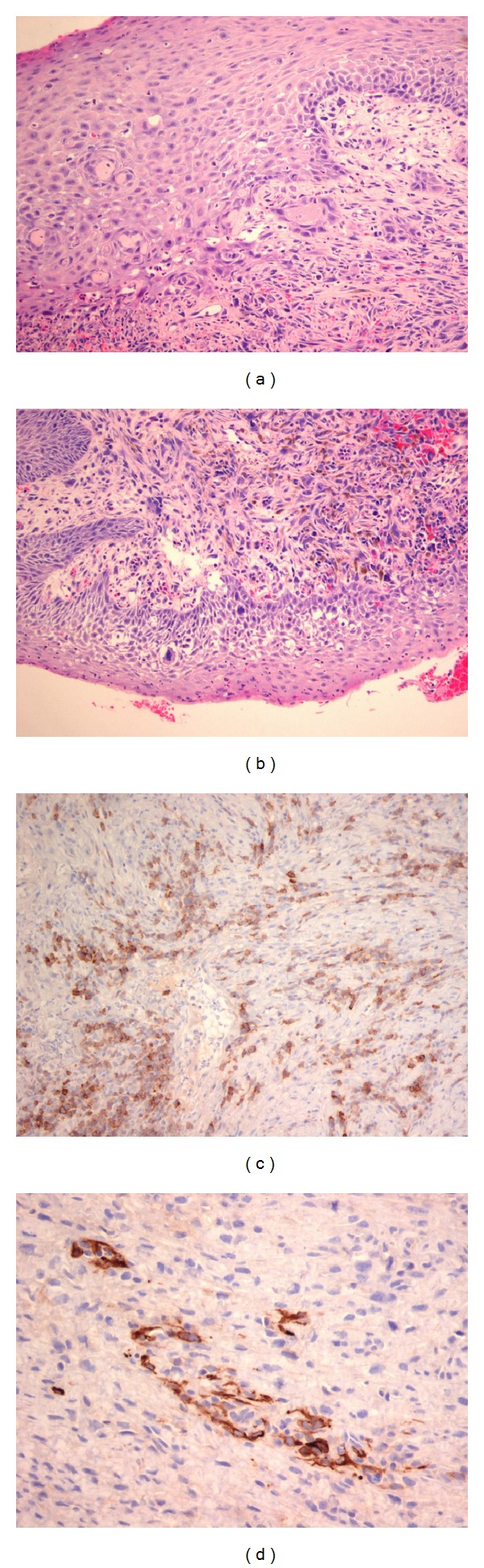
Definitive histological examination showing a carcinosarcoma with carcinomatous and sarcomatous components. Immunohistochemistry with monoclonal antibodies (anticytokeratin CK7, CK 8–18, and CK 5-6) for actin, P63, vimentin, S100, desmin, and EMA (epithelial membrane antigen) has been performed. The tumour showed strong positivity for vimentin, EMA, and focal elements for CK 8–18. (a) Hematoxylin-eosin (20x). “In situ” squamous cell carcinoma with cells infiltrating the subepithelial corium. Lower right: undifferentiated spindle cell carcinoma cells. (b) Hematoxylin-eosin (20x). “In situ” squamous cell carcinoma with atypical cells at the basal layers, a sarcomatous component, and hemosiderin deposits. (c) EMA (20x). Immunohistochemistry: carcinomatous cells with membrane positivity for EMA. (d) CK 8–18 (40x): focal positivity for cytokeratin 8–18.

The definitive histological examination disclosed a carcinosarcoma of the entire epiglottis laryngeal side (including the petiole) (Figure 1) with disease-free margins (pT2N0 M0, Stage II).

The postoperative course was uneventful. At 3 weeks after surgery the patient was evaluated by the multidisciplinary team and considering the histological definitive diagnosis no indications for further treatments were proposed.

A tracheoplasty was performed after 3 weeks as laryngoscopy revealed regular surgical outcomes with conserved glottic space.

At the moment the patient is followed up regularly, she is disease-free at 12 months after surgery, and she is showing good functional results and a good quality of life.

## 3. Discussion and Conclusion

Carcinosarcoma is a rare malignant tumour with both carcinomatous and sarcomatous components. It is difficult to assess the true incidence of this pathological entity, even if it has been estimated that carcinosarcoma of larynx and hypopharynx represents less than 1% of all malignant tumours [6], and both sexes are equally affected.

About its histogenesis, some hypotheses have been proposed as carcinosarcoma could be considered as (1) a carcinoma with a reactive mesenchymal component; (2) a tumour in which carcinoma and sarcoma develop simultaneously and independently; (3) a tumour arising from embryonic epithelial and mesenchymal residual tissues; (4) a malignancy arising from a totipotent malignant cell that therefore differentiates into carcinomatous and sarcomatous components [10–12].

According to the World Health Organization (WHO) classification [12], three different subtypes of malignant mixed tumours can be identified: carcinoma ex-pleomorphic adenoma, metastasizing mixed tumor, and carcinosarcoma [13, 14]. In carcinoma ex-pleomorphic adenoma, the epithelial component only becomes malignant (thus developing an adenocarcinoma), while in metastasizing mixed tumor and carcinosarcoma, or true malignant mixed tumor, there is a dual malignant component (carcinomatous and sarcomatous) which are therefore considered biphasic [15, 16].

Concerning the localization of carcinosarcomas, major salivary glands are the most frequent sites reported in head and neck, whereas other sites such as nasal and oral cavity, nasopharynx, bronchus, lung, and trachea are rare, and larynx is considered even more rare [1–5, 17]. The clinical presentation of laryngeal carcinosarcoma is similar to that of other laryngeal carcinomas [18], with dysphonia, dyspnoea, and dysphagia as the most frequent symptoms reported.

There is no consensus about the best treatment options for carcinosarcoma; however surgical excision with wide margins is the most recommended treatment [17, 18]. The specific therapeutical approach should be anyway tailored to the tumor stage, localization, and size.

In the case presented a supraglottic horizontal laryngectomy was performed endoscopically with laser CO(2). It has already been reported that the effectiveness of endoscopic laser horizontal laryngectomy is similar to the external approach in terms of oncological outcome (as the surgical margins were disease-free) and functional results, in case of supraglottic laryngeal squamous cell carcinoma [19]. However to our knowledge this is the first case of laryngeal carcinosarcoma that has been treated endoscopically. We decided not to proceed with a prophylactic neck dissection, due to (i) the general health problems of the patient, in particular the high neck vascular impairment (that represented a strong contraindication to a neck dissection), (ii) the laryngeal cancer stage (T2NOMO), and (iii) the definitive histologic diagnosis.

However, the patient is under strict clinical (endoscopic) and radiologic (echographic and/or CT-PET) followup and presently is without evidence of disease.

On radiotherapy there is no consensus in the literature as the mesenchymal component is reported to be resistant to irradiation [12].

The prognosis of carcinosarcoma is also controversial [12], and it is reported to be worse than that of squamous cell laryngeal carcinoma [12]. Nonetheless, due to the extraordinarily rare incidence of this tumor, it will be essential to acquire more information on its biological behaviour, so that appropriate treatment modalities can be defined.

## References

[1] Sanabre AA, Gonzalez-Lagunas J, Redecilla PH, Martin GR (1998). Carcinosarcoma of the maxillary sinus: a case report. *Journal of Oral and Maxillofacial Surgery*.

[2] Pang PCW, To EWH, Tsang WM, Liu TL (2001). Carcinosarcoma (malignant mixed tumor) of the parotid gland: a case report. *Journal of Oral and Maxillofacial Surgery*.

[3] Bhalla RK, Jones TM, Taylor W, Roland NJ (2002). Carcinosarcoma (malignant mixed tumor) of the submandibular gland: a case report and review of the literature. *Journal of Oral and Maxillofacial Surgery*.

[4] Klijanienko J, Vielh P, Duvillard P, Luboinski B (1992). True carcinosarcoma of the larynx. *Journal of Laryngology and Otology*.

[5] Srinivasan U, Talvalkar GV (1979). True carcinosarcoma of the larynx: a case report. *Journal of Laryngology and Otology*.

[6] Luna-Ortiz K, Mosqueda-Taylor A (2006). Supracricoid partial laryngectomy as a primary treatment for carcinosarcoma of the larynx. *Ear, Nose and Throat Journal*.

[7] Madrigal FM, Godoy LMR, Daboin KP, Casiraghi O, Garcia AM, Luna MA (2002). Laryngeal osteosarcoma: a clinicopathologic analysis of four cases and comparison with a carcinosarcoma. *Annals of Diagnostic Pathology*.

[8] Veivers D, De Vito A, Luna-Ortiz K, Brasnu D, Laccourreye O (2001). Supracricoid partial laryngectomy for non-squamous cell carcinoma of the larynx. *Journal of Laryngology and Otology*.

[9] Alexander A, Walter N, Varghese A, Mary L (2007). Pseudosarcoma: a diagnostic and treatment dilemma. *Journal of Postgraduate Medicine*.

[10] Wick MR, Swanson PE (1993). Carcinosarcomas: current perspectives and an historical review of nosological concepts. *Seminars in Diagnostic Pathology*.

[11] Ernster JA, Franquemont DW, Sweeney JP (2000). Initial report of a case of carcinosarcoma of the supraglottis. *Ear, Nose & Throat Journal*.

[12] Zhang M, Zhao L-, Li X-M, Zhou L, Lin L, Wang S-Y (2013). True carcinosarcoma of the larynx. *Journal of Laryngology and Otology*.

[13] Barnes L, Eveson J, Reichart PA, Sidransky D, Kleihues P, Sobin LH (2005). Lymphoepithelial carcinoma. *World Health Organization Classification of Tumors: Pathology and Genetics of Head and Neck Tumors*.

[14] Nistal M, Yébenes-Gregorio L, Esteban-Rodríguez I, Bernáldez R, Regadera J (2005). Malignant mixed tumor of the larynx. *Head and Neck*.

[15] Gnepp DR (1993). Malignant mixed tumors of the salivary glands: a review. *Pathology Annual*.

[16] Furukawa M, Suzuki H, Matsuura K, Takahashi E, Suzuki H, Tezuka F (2001). Carcinoma ex pleomorphic adenoma of the palatal minor salivary gland with extension into the nasopharynx. *Auris Nasus Larynx*.

[17] Stomeo F, Rocca PC, Bozzo C, Bianchini C, Meloni F, Pastore A (2009). Laryngeal true malignant mixed tumor. *Head and Neck*.

[18] Ianniello F, Ferri E, Armato E, Longo L, Zambenedetti P (2001). Carcinosarcoma of the larynx: immunohistochemical study, clinical considerations, therapeutic strategies. *Acta Otorhinolaryngologica Italica*.

[19] Marioni G, Bottin R, Staffieri A, Altavilla G (2003). Spindle-cell tumours of the larynx: diagnostic pitfalls: a case report and review of the literature. *Acta Oto-Laryngologica*.

